# Analyzing audience participation at the 43rd workshop of the german surgical working group on endocrinology (CAEK): gender balance in academic discussion dynamics in endocrine surgery

**DOI:** 10.1007/s00423-026-04176-2

**Published:** 2026-08-07

**Authors:** Anna Mikl, M. Kießler, M. Berlet, M. Martignoni, H. Friess, C. Chiapponi

**Affiliations:** School of Medicine and Health, Department of Surgery, Technical University of Munich, TUM University Hospital, Munich, Germany

**Keywords:** Endocrine surgery, Gender differences, Presentation habits, Questioner behaviors

## Abstract

**Introduction:**

Although many surgical fields remain male dominated, the rising number of female medical students and physicians raises important questions about engagement dynamics within academic and professional settings. Endocrine Surgery (ES) currently demonstrates high rates of female representation among its fellows all over the world. This study examines gender differences in audience participation at the 43rd Workshop of the Surgical Working Group on Endocrinology (CAEK).

**Methods:**

Lecture sessions of the 43rd Workshop of the CAEK were prospectively observed and analyzed. The study recorded the gender appearance of chairs, speakers, participants asking the first question following each lecture and whether this influenced subsequent audience participation. Gender distributions among attendees and question patterns were compared.

**Results:**

A total of 385 participants (52% female, 48% male) attended the conference. 37 sessions were included. Overall, 63 questions were recorded. 34 (54%) came from women and 29 (46%) from men.

The first question was posed by a male participant in 14 sessions (51.9%) and by a female participant in 13 sessions (48.1%).

When discussion was started by a man, 65.9% of subsequent questions came from other men. 90.9% of follow-up questions came from women, when discussion was opened by another woman.

**Conclusion:**

Female participation at the 43rd Workshop CAEK was well balanced, although the gender of the first questioner influenced subsequent audience engagement, as observed in other studies. These findings suggest equitable academic engagement in endocrine surgery in Germany, while indicating that the gender of the first questioner is associated with subsequent audience participation.

## Introduction

Over the past two decades, the gender composition of the medical workforce has undergone a substantial transformation. In Germany, women now represent a clear majority of medical students, accounting for approximately 60–65% of enrolments in human medicine - a proportion that has remained stable in recent years and mirrors trends observed across most high-income countries [1]. 

This sustained feminization of the medical pipeline ensures that the future physician workforce will, at the point of entry into professional training, be predominantly female.

Despite this demographic shift, surgical specialties in Germany remain strongly male-dominated. Women continue to be underrepresented in surgical training programs and more markedly so at senior and leadership levels. Depending on the subspecialty, female surgeons account for only 15–25% of board-certified surgeons, with particularly low proportions in general surgery, visceral surgery, and orthopedic surgery [2]. This discrepancy illustrates a persistent ‘leaky pipeline,’ whereby women enter medical school in high numbers but are lost disproportionately along the pathway to surgical careers - a structural concern with direct implications for workforce sustainability in the German healthcare system.

Within this context, endocrine surgery represents a notable exception. Several international and European studies suggest that endocrine surgery harbors a comparatively higher proportion of female surgeons than most other surgical subspecialties, with female representation estimated between 35% and 58% depending on career stage [3]. In Germany, endocrine surgery is predominantly embedded within visceral surgery but is characterized by a distinct academic culture and a pronounced emphasis on specialized training and clinical research. Whether this relative gender diversity reflects structural or cultural features specific to the subspecialty - and whether it translates into more equitable academic participation - remains incompletely understood.

Academic engagement and research productivity are central determinants of career progression in German academic medicine. Advancement to senior positions typically requires a strong publication record, competitive research funding, and visible participation in the scientific community [4]. 

Conferences serve as critical platforms for presenting research, networking, and contributing to scholarly discourse. Participation in academic discussions - particularly question-asking following presentations - enhances professional visibility and signals research involvement, which is especially relevant in competitive surgical environments.

Prior research across medical and scientific conferences has consistently demonstrated gender differences in visible academic participation: women are less likely than men to ask questions following presentations, even when equally represented among attendees [5, 6]. Qualitative studies suggest that lower self-confidence, fear of negative evaluation, and the absence of senior female role models contribute to this behavior [4, 7]. In male-dominated fields such as surgery, reduced visibility of women in academic discussions has been proposed to influence perceptions of professional belonging and academic visibility. The gender of the first questioner, in particular, has been reported to shape the gender composition of subsequent discussion – a gender-matching effect – although this has rarely been quantified in surgical settings [8, 9]. 

Against this background, examining gender-specific participation patterns at a conference in a subspecialty with relatively high female representation offers a unique opportunity to assess whether a more gender-diverse environment translates into more equitable academic engagement. The present study investigates gender differences in question-asking behavior at the 43rd Workshop of the German Surgical Working Group on Endocrinology (CAEK) at Leipzig 2025, with particular focus on the influence of the first questioner’s gender on subsequent discussion dynamics and the role of moderator gender in shaping audience participation.

## Materials and methods

### Study design and setting

This was a prospective, observational study conducted at the 43rd Workshop of the German Surgical Working Group on Endocrinology (CAEK), held in Leipzig in 2025. Before starting this study an official request was sent out to the organizers of the workshop, which was accepted. All 37 scheduled lecture sessions were observed. Each observed unit corresponded to a single scheduled lecture of up to 15 min in duration, delivered by one presenting speaker and including its dedicated discussion period; accordingly, the term ‘session’ is used here to denote an individual lecture rather than a thematic block comprising several presentations. Because a given moderator frequently chaired more than one consecutive lecture, moderator gender was recorded separately for each lecture, and the number of presenting speakers therefore equals the number of lectures (*n* = 37). Eligible sessions were required to be open to all conference participants and to fulfil predefined inclusion criteria. Round-table discussions, interactive workshops, and sessions without a designated discussion period were excluded. All included sessions had a maximum total duration of 15 min, inclusive of the question-and-answer period.

### Data Collection

The following variables were recorded for each session: the perceived gender of the presenting speaker, of the session moderator, and of the audience member posing the first question; the total number of subsequent questions by gender; and the total number of comments by gender. Gender assignment was based on the participant’s name, visual appearance, and voice. The same observational construct - perceived gender inferred from name, visual appearance, and voice - was applied consistently to speakers, moderators, and audience members; no self-reported gender information was collected, and all three roles were assessed under an identical definition. In cases of uncertainty, participants would have been coded as ‘unknown’; however, this did not occur, and all contributions could be unambiguously assigned to either the male or female category. Non-binary or gender-diverse identities were not recorded as a distinct category, representing a limitation of the applied approach to gender classification.

Questions were categorized into four predefined groups based on their primary orientation: topic - addressing the general subject of the presentation; type - reflecting the framing or methodological approach; focus - relating to a specific patient case; or ‘not classifiable’. Question valence was coded on a three-point scale: positive (+ 1), neutral (0), or negative (-1). Comments were similarly categorized as ‘patient case,’ ‘different treatment options,’ ‘other,’ or ‘not classifiable.’ If a participant posed multiple distinct questions to the same speaker, each was counted separately; clarification follow-ups to the same initial question were not counted. Statements without interrogative intent were recorded separately as comments and excluded from question-specific analyses. All classifications of question content and valence, and of comment category, were performed in real time during each lecture by a single trained observer applying the predefined coding scheme described above; no post hoc re-coding or independent second rating was undertaken.

### Statistical analyses

All statistical analyses were performed using Python (SciPy v1.11) [10]. Categorical variables were summarized as frequencies and proportions. Gender distributions in question-asking were compared using Pearson’s chi-square tests. Where expected cell frequencies fell below five, Fisher’s exact test was applied. A two-tailed binomial test was used to assess whether the proportion of male versus female first questioners deviated significantly from a 50:50 distribution. To examine whether the gender of the first questioner influenced subsequent audience participation, a 2 × 2 contingency table was constructed and analyzed using both chi-square and Fisher’s exact tests. The same approach was applied to assess the effect of moderator gender on the gender distribution of questions. A p-value < 0.05 was considered statistically significant for all tests. No correction for multiple comparisons was applied in this exploratory analysis.

## Results

### Conference overview and participant characteristics

**Fig. 1:**
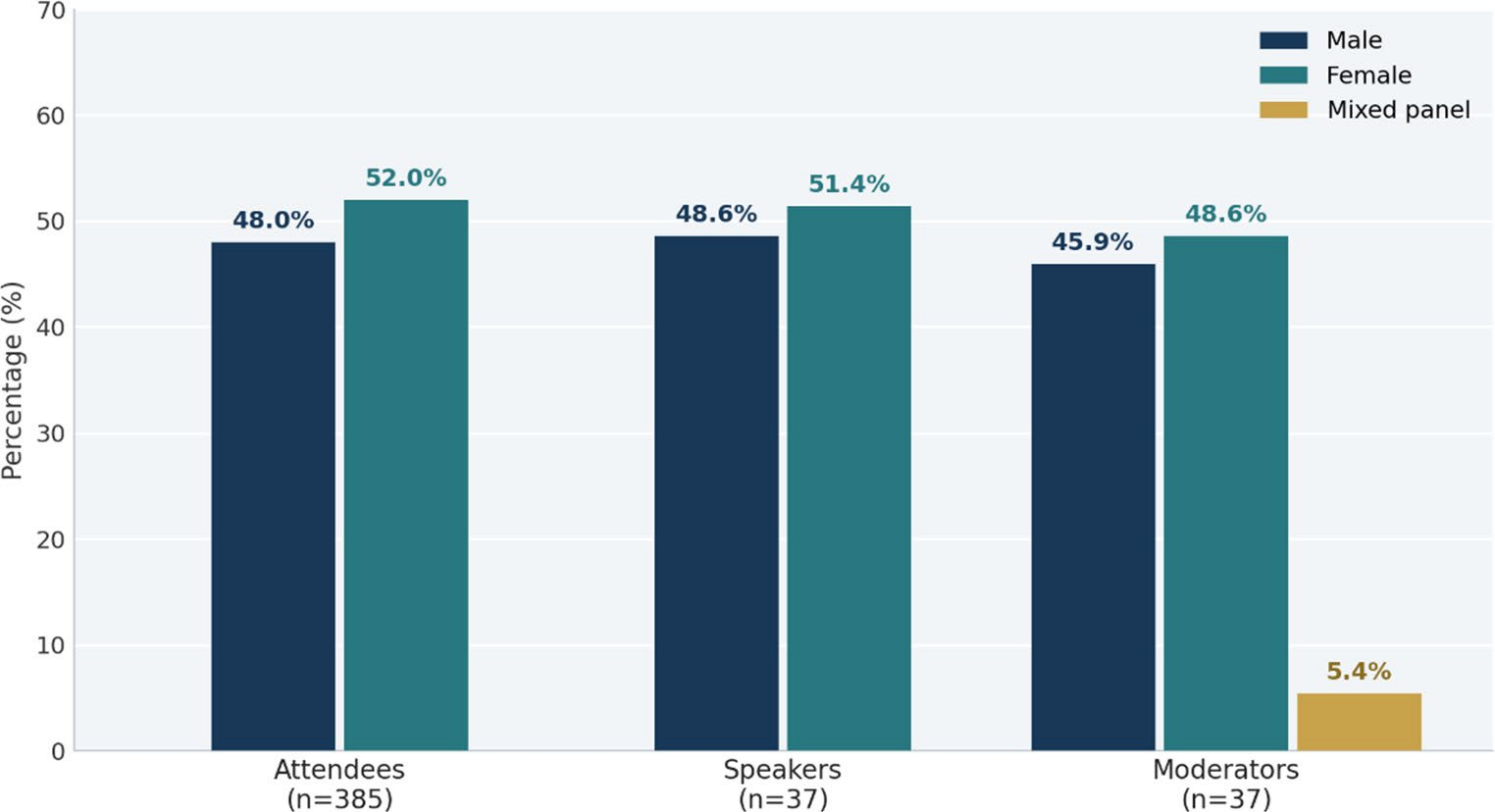
Gender distribution across conference roles (*n* = 385 attendees; *n* = 37 sessions)

A total of 385 participants attended the 43rd CAEK Workshop, of whom 200 (52.0%) were female and 185 (48.0%) were male. Across 37 sessions, speakers were nearly equally distributed by gender (male: *n* = 18, 48.6%; female: *n* = 19, 51.4%). Moderators were similarly balanced (male: *n* = 17, 45.9%; female: *n* = 18, 48.6%; mixed panel: *n* = 2, 5.4%). The gender distribution across conference roles is summarized in Fig. 1. Of 37 sessions, 27 (73.0%) included at least one documented audience question; 10 sessions (27.0%) concluded without audience discussion.

### First questioner gender and subsequent discussion dynamics

**Fig. 2:**
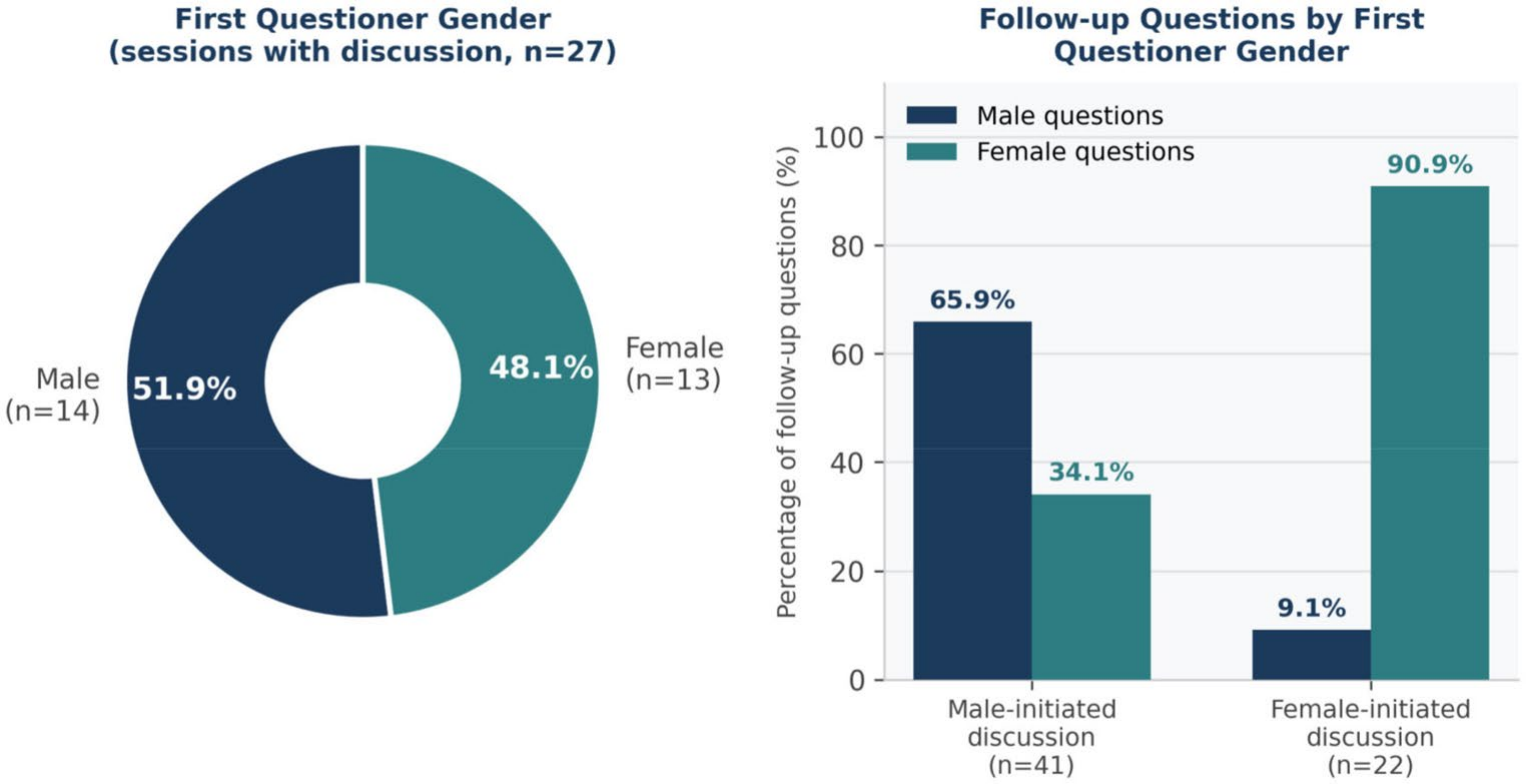
Gender distribution of first questioners across sessions with discussion (*n* = 27). Right: Gender distribution of follow-up questions stratified by the gender of the first questioner

Among the 27 sessions with audience discussion, the first question was posed by a male participant in 14 sessions (51.9%) and by a female participant in 13 sessions (48.1%). This distribution did not deviate significantly from an equal split (chi-square: χ²=0.037, *p* = 0.847; binomial test: *p* = 1.000; Fig. 2, left panel).

The gender of the first questioner, however, exerted a strong and statistically significant influence on subsequent audience participation. In sessions where a male participant asked the first question, 65.9% (*n* = 27) of follow-up questions were posed by men and 34.1% (*n* = 14) by women. In contrast, when a female participant initiated the discussion, 90.9% (*n* = 20) of follow-up questions were posed by women and only 9.1% (*n* = 2) by men (Fisher’s exact test: OR = 19.29, *p* < 0.001; chi-square: χ²=16.355, df = 1, *p* < 0.001; Fig. 2, right panel). This finding provides strong evidence for a gender-matching or role-model effect in academic discussion dynamics.

### Total questions and comments by gender

**Fig. 3:**
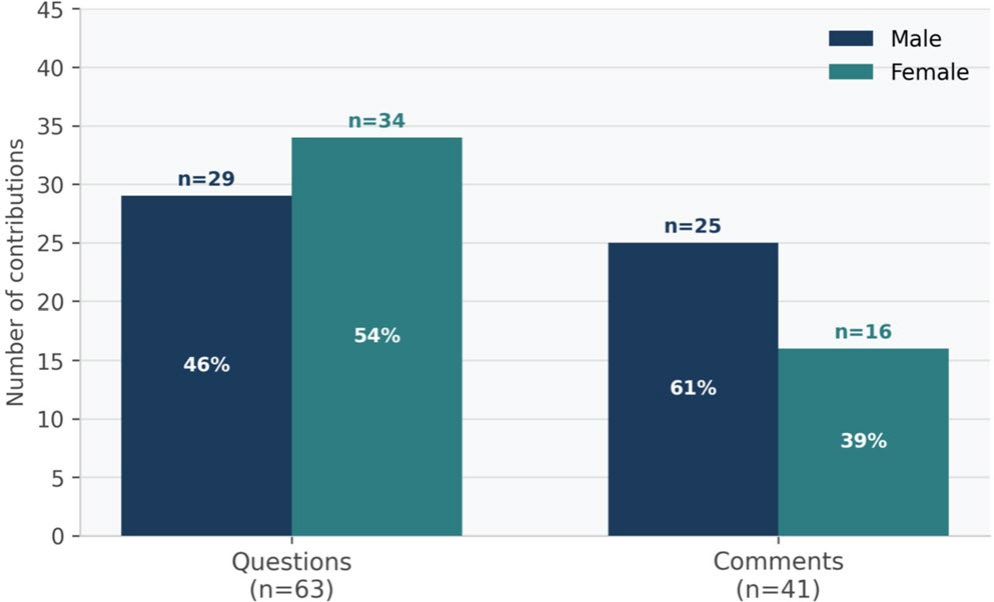
Total number of audience questions (*n*=63) and comments (*n*=41) by gender

A total of 63 audience questions were recorded across all sessions. Women posed 34 questions (54.0%) and men 29 (46.0%). This difference did not reach statistical significance (chi-square: χ²=0.397, *p* = 0.529; Fisher’s exact test: OR = 1.08, *p* = 0.787). A total of 41 comments were recorded, of which 25 (61.0%) were made by men and 16 (39.0%) by women. This difference was also not statistically significant (chi-square: χ²=1.976, *p* = 0.160). Notably, women asked more questions overall despite men providing more comments. (Fig. 3).

### Qualitative characteristics of questions and comments

**Fig. 4:**
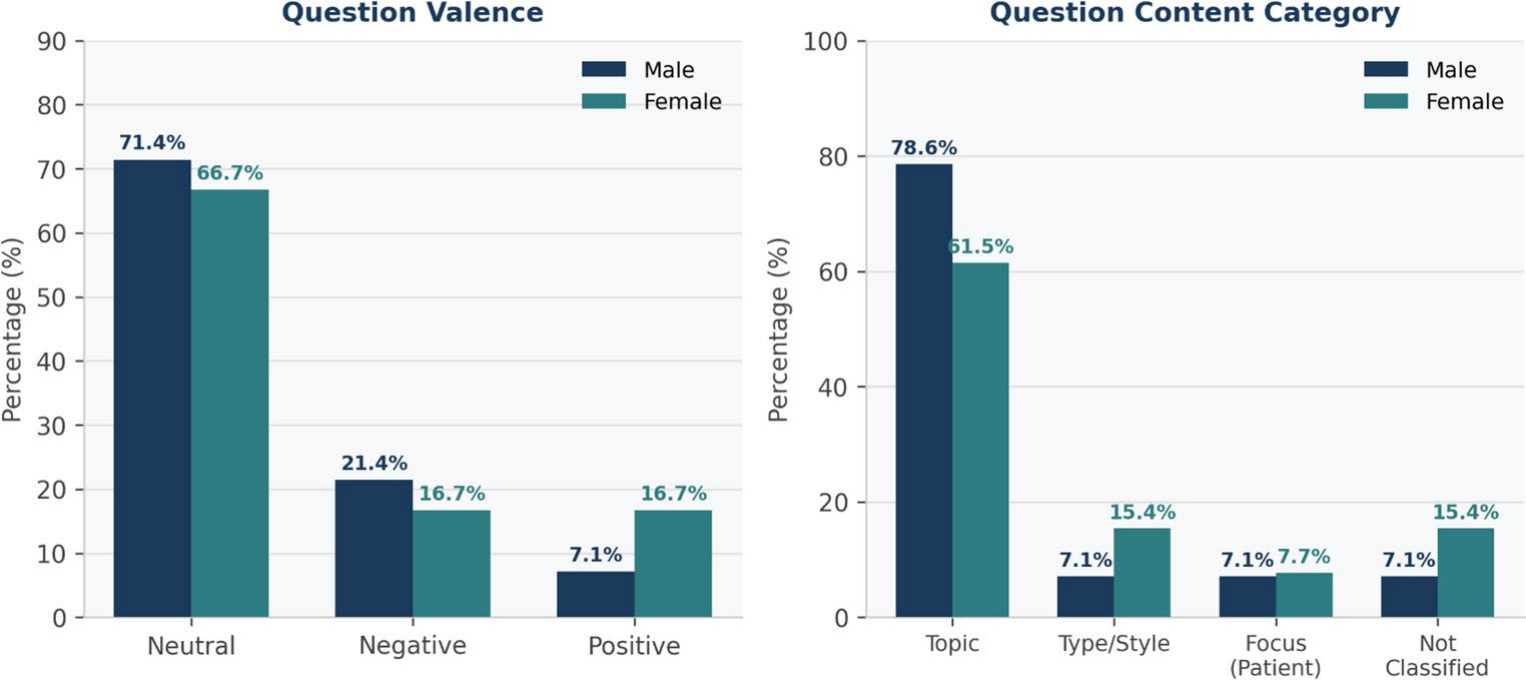
Qualitative characteristics of first questions by gender. Left: valence distribution (neutral, negative, positive). Right: content category distribution

No significant qualitative differences were identified between male and female questioners in terms of question valence or content category (Fig. 4). The majority of first questions across both genders were topic-oriented (male: 11/14, 78.6%; female: 8/13, 61.5%). Questions framed positively, negatively, or neutrally were similarly distributed: among male questioners, 10 of 14 (71.4%) were neutral, 3 (21.4%) negative, and 1 (7.1%) positive; among female questioners, 8 of 12 (66.7%) were neutral, 2 (16.7%) negative, and 2 (16.7%) positive. These distributions did not differ significantly between genders.

### Moderator gender and question patterns

**Fig. 5:**
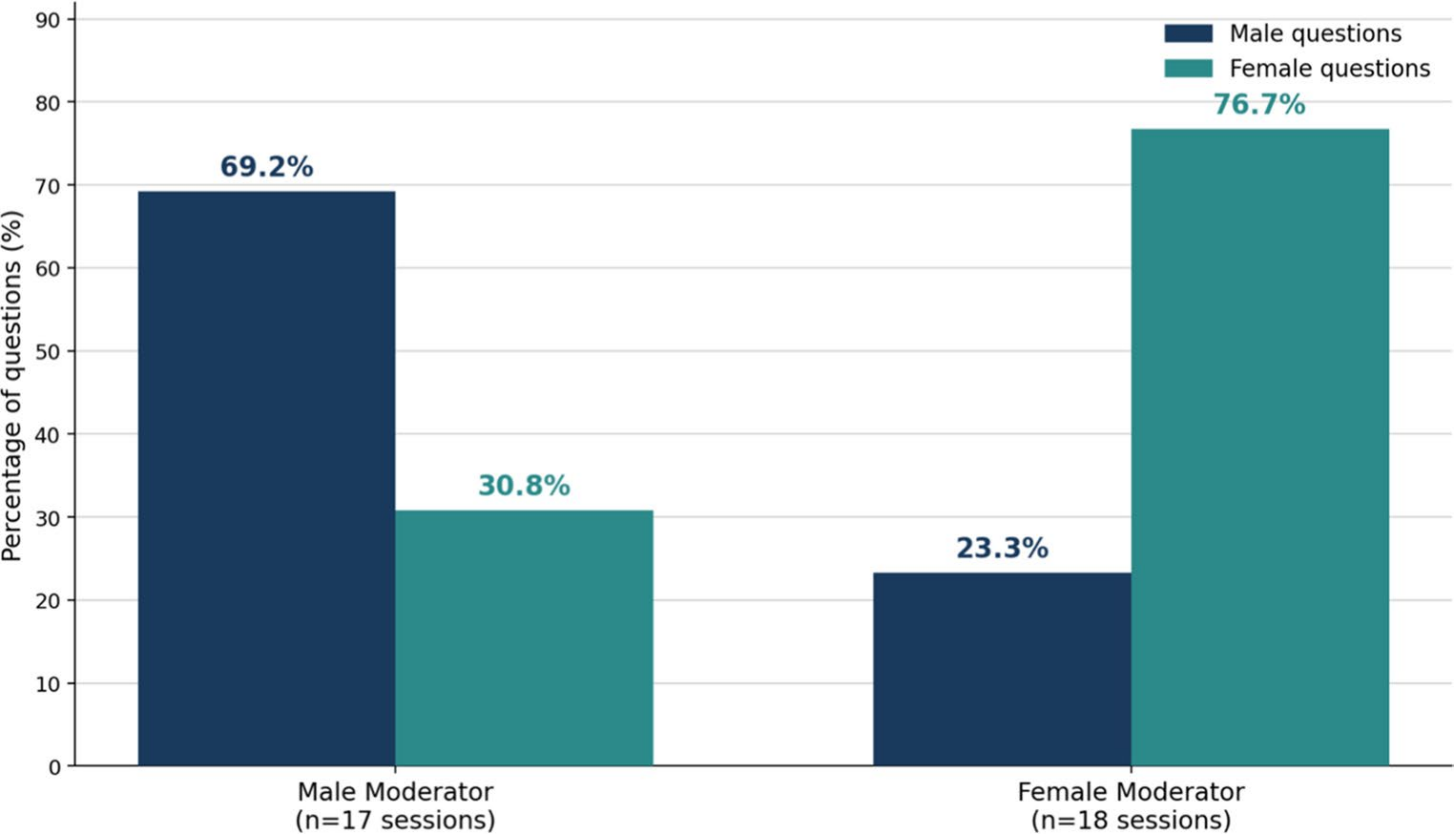
Gender distribution of audience questions stratified by moderator gender

Moderator gender was significantly associated with the gender distribution of audience questions. In sessions chaired by male moderators (*n* = 17 sessions), 18 of 26 questions (69.2%) were posed by men. In sessions chaired by female moderators (*n* = 18 sessions), 23 of 30 questions (76.7%) were posed by women (Fisher’s exact test: OR = 7.39, *p* = 0.001; chi-square: χ²=10.088, df = 1, *p* = 0.0015; Fig. 5). Moderator gender was not significantly associated with the gender of the first questioner (Fisher’s exact: OR = 4.50, *p* = 0.115).

**Fig. 6:**
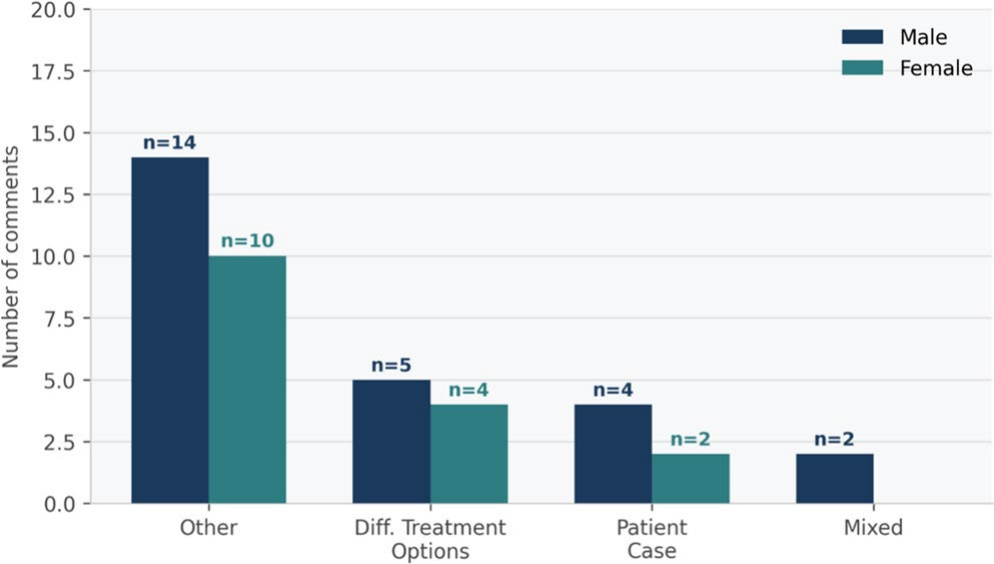
Comment content distribution by gender. Men contributed a greater absolute number of comments (*n* = 25 vs. *n* = 16)

As seen in Fig. 6, comment content was distributed across four categories: ‘other’ (n = 12), ‘different treatment options’ (n = 6), ‘patient case’ (n = 5), and ‘mixed’ (*n* = 1). Men contributed more comments overall (*n* = 25, 61.0%), whereas women predominated in question-asking (*n* = 34, 54.0%).

### Summary of statistical results

Table 1.

**Table 1:** Summary of conference participation and statistical results

Variable	Male *n* (%)	Female *n* (%)	Statistical test	*p*-value
Attendees	185 (48.1%)	200 (52.0%)	—	—
Speakers	18 (48.6%)	19 (51.4%)	—	—
Moderators*	17 (45.9%)	18 (48.6%)	—	—
First questioner (of 27 sessions)	14 (51.9%)	13 (48.1%)	χ²=0.037 Binomial test	*p* = 0.847 *p* = 1.000
Total questions (*n* = 63)	29 (46.0%)	34 (54.0%)	χ²=0.397 Fisher’s exact OR = 1.08	*p* = 0.529 *p* = 0.787
Total comments (*n* = 41)	25 (61.0%)	16 (39.0%)	χ²=1.976	*p* = 0.160
Follow-up questions: male-initiated sessions	27 (65.9%)	14 (34.1%)	Fisher’s OR = 19.29 χ²=16.355	*p* < 0.001 *p* < 0.001
Follow-up questions: female-initiated sessions	2 (9.1%)	20 (90.9%)	Fisher’s OR = 19.29 χ²=16.355	*p* < 0.001 *p* < 0.001
Questions in male moderator sessions	18 (69.2%)	8 (30.8%)	Fisher’s OR = 7.39 χ²=10.088	*p* = 0.001 *p* = 0.0015
Questions in female moderator sessions	7 (23.3%)	23 (76.7%)	Fisher’s OR = 7.39 χ²=10.088	*p* = 0.001 *p* = 0.0015

*Moderator roles comprised 17 male-chaired (45.9%) and 18 female-chaired (48.6%) lectures shown above, together with 2 lectures (5.4%) chaired by mixed-gender panels, giving 37 lectures in total and matching the counts in Fig. 1

## Discussion

This prospective observational study examined gender differences in question-asking behavior and discussion dynamics at the 43rd CAEK Workshop within the broader context of a rapidly feminizing medical workforce. The principal findings are threefold: overall audience participation was gender-balanced, with women asking slightly more questions than men in aggregate; the gender of the first questioner exerted a strong and statistically significant influence on subsequent discussion - constituting a robust gender-matching effect (OR = 19.29, *p* < 0.001); and moderator gender was independently associated with the gender distribution of questions (OR = 7.39, *p* = 0.001).

The observation that women asked the majority of total questions (54.0%) at a surgical conference challenges assumptions prevalent in prior literature that women are inherently less willing to participate in public academic forums [5, 6]. In most previously studied medical and scientific conference settings, women underparticipate relative to their attendance proportion. The comparatively high female representation at CAEK - among both speakers and moderators - may have created an environment in which participation norms are less asymmetric, consistent with the hypothesis that endocrine surgery’s gender composition may offer transferable structural lessons.

The gender-matching effect identified in this study is among the most striking findings. When a female participant asked the first question, 90.9% of all subsequent questions were posed by women, with near-complete withdrawal of male participation. Conversely, male-initiated discussions maintained a more balanced but male-leaning distribution. This asymmetry - quantified here with high statistical precision (OR = 19.29, *p* < 0.001) - suggests that the identity of the first speaker in a discussion exerts a disproportionate influence on subsequent group dynamics. Such gender-matching mechanisms have been theorized in organizational psychology and educational research but have rarely been quantified with this level of specificity in a surgical conference setting. A comparable pattern, in which a female first questioner was followed by a substantially higher proportion of female questioners, has previously been reported for university research seminars [8].

The significant association between moderator gender and question distribution (OR = 7.39, *p* = 0.001) represents a novel finding. Sessions chaired by female moderators yielded substantially higher rates of female question-asking (76.7% vs. 30.8% in male-moderated sessions), independent of the gender of the first questioner. This finding supports the notion that moderators serve not only as procedural facilitators but also as implicit role models whose gender and behaviour may be associated with audience engagement patterns. The gender composition of session chairs was therefore associated with the gender distribution of audience participation. Because scientific sessions are frequently chaired by teams of two or more moderators, often of mixed gender, the extent to which associations observed for single-gender chairs transfer to these more common formats remains uncertain and needs dedicated investigation.

The absence of qualitative differences in question content or valence between male and female participants is important. It confirms that observed disparities in academic participation are quantitative in nature—relating to frequency and dynamics—rather than qualitative, and that women’s contributions are equally substantive, patient-oriented, and academically rigorous. This finding challenges persistent assumptions about gender-based differences in scientific communication style.

Men contributed more comments overall (61.0%), whereas women predominated in question-asking (54.0%). This divergence may reflect different socialization patterns or strategic preferences in academic communication: comments tend to be less formal, shorter, and more reflexive, whereas questions are more structured and publicly visible. Whether this pattern reflects genuine communicative preference or differential comfort with high-visibility contribution modes warrants further investigation.

Several limitations should be acknowledged. First, gender assignment was based on observable biological characteristics and did not account for non-binary or gender-diverse identities, potentially introducing misclassification bias and failing to represent the full spectrum of gender identity. Second, data on professional rank, seniority, institutional affiliation, and prior familiarity with speakers were unavailable, and these factors may substantially influence participation behavior, particularly with respect to initiating discussion. Third, the study was conducted at a single specialty conference with comparatively high female representation, which limits generalizability to more male-dominated surgical fields. Fourth, the observational design precludes causal inference: observed associations between moderator or first questioner gender and subsequent participation may be confounded by unmeasured session-level variables. Finally, the sample of 37 sessions and 63 total questions, while sufficient to detect the observed effect sizes, limits the statistical power for subgroup analyses. In addition, question content, valence, and comment categories were coded in real time by a single observer, without independent duplicate coding or a formal assessment of inter-rater reliability, which may limit the reproducibility of the qualitative classifications. Finally, moderator effects were examined at the level of individual chairs; sessions led by mixed-gender panels were too few (*n* = 2) to be analysed separately, so the influence of mixed-gender chairing - a common format at larger meetings –- could not be assessed.

## Conclusion

This study demonstrates that gender differences in academic conference participation are driven not by disparities in engagement, expertise, or quality of contributions, but by the social dynamics governing who speaks first and who chairs the session. The first question was posed by men and women in a comparable number of sessions (14 versus 13; χ²=0.037, *p* = 0.847), and women asked slightly more questions overall (54.0%), indicating substantial engagement once discussion was underway. The gender of the first questioner emerged as a critical determinant of subsequent audience participation, with a strong gender-matching effect quantified at OR = 19.29 (*p* < 0.001). Moderator gender independently predicted the gender distribution of questions (OR = 7.39, *p* = 0.001), highlighting an association between the gender of session chairs and the observed distribution of audience participation.

These findings are descriptive and should be interpreted with caution. They indicate that, in a subspecialty in which speakers, chairs, and attendees were present in near-equal proportions, audience participation was likewise balanced. Where equitable academic visibility is an explicit objective, the present data are consistent with the view that conference roles - speakers, chairs, and discussants - could be allocated in proportions that reflect the composition of the specialty. Whether such proportionate representation causally improves participation, and how it interacts with the mixed-gender chairing formats common at larger meetings, cannot be established from an observational study of this size. Future research across multiple disciplines, conference formats, and professional contexts is warranted to further characterize these dynamics and to characterize these dynamics across disciplines and conference formats.

## Data Availability

No datasets were generated or analysed during the current study.
